# The Medical Department of the University of Texas

**Published:** 1887-03

**Authors:** 


F. E. DANIEL, M. D., EDITOR AND PUBLISHER.

COLLABORATORS:

*E. J. Doering, M. D., Chicago.*
*Geo. Cupples, M. D., San Antonio.*
*Odo Betz, M. D., Germany.*
*E. J. Beall, M. D., Fort Worth, Texas.*
*T. C. Osborn, M. D., Cleburne, Texas.*
*Wm. Penny, M. D., New York.*
*H. O. Marcy, M. D., Boston.*
*C. K. Gregg, M. D., Mexico.*
*R. M. Swearingen, M. D., Austin.*
*C. H. Wilkinson, M. D., Galveston.*
*J. W. McLaughlin, M. D., Austin.*
*R. H. L. Bibb, M. D., Mexico.*

### THE MEDICAL DEPARTMENT OF THE UNIVERSITY OF TEXAS.

The City Council of Galveston, through a committee of resident physicians, consisting of Drs. J. F. Y. Paine, J. M. Haden, H. A. West, C. W. Trueheart and H. P. Cook, have memoralized the Governor, and the Legislature now in session, to take some action "by bill or otherwise, to carry out the provisions of the law authorizing the location and establishment of the Medical Department of the University" in that city.

The memorial sets forth 1st; that according to experience of educators the interest of the Medical, is incompatible with that of other branches—the proximity of a hospital being an essential requisite for the former, would be a horror to the latter, which would operate to keep academic students away. The memorialists assert that this view is almost generally taken of the subject, both in this county and in Europe, and that accordingly, in most successful colleges, they are separate—if not in separate towns, at least in separate buildings; and cite well known instances. In London the Medical Schools and the University are entirely separate and disconnected, and each Hospital has its Medical School.

2nd, That the history of Medical Schools which have formed "integral parts of Universities, and have been more or less organically connected and under one general head or government"—and where medicine is taught theoretically, but not clinically, have not succeeded in turning out doctors, however thorough the scholastic training may have been; that this is appreciated by students and shown in the relatively small annual matriculation in the medical department, compared with that of scnools connected with large hospitals. Illustrations are given as follows :

At Yale, ("harmonious, symmetrical university" i. e. altogether) —the catalogue for '86–7, gives a summary of 1,134 students, of whom only 27 were medical matriculants. The University of Virginia, (ditto ditto) gives a yearly average of 342 students with only 46 in the medical department; whereas, in the medical department of the University of Pennsylvania, (separate, and with hospital) there were 386 students. Medical Department University City New York, nearly 600; ditto, University Louisiana, 250, &c. Jefferson Medical College (which is the medical branch of Washington College situated at Cannonville, Pa.) 500, &c. &c.

In view of these facts, "it is difficult," the memorlalists say, "to avoid the conclusion that the success of the Medical Department of the University of Texas, depends in a large degree upon its separate organization," and that its location at Galveston "is simply a matter of expediency, and does not imply a severance from the main University; that it should be under the control of a common board of Regents."

Conceding then the essentiality of clinical instruction, the memorialists proceed to show the advantages possessed by Galveston over any other Texas city in the matter of supply of clinical and anatomical material, and make a very excellent showing; for instance, in 1883 and 1886 (we take these years as showing the highest and the lowest numbers in the table), there were treated in St. Mary's and the Marine Hospital an aggregate of 2948 (for '83) and 2114 (for '86) patients, embracing every variety of disease and injury (except of course epidemic or infectious diseases.) Of the 2114 cases last year, 296 were surgical, including fractures, dislocations and every variety of wounds, surgical diseases of bones, joints, blood-vessels, viscera, skin, genital organs, &c. When the new Sealey Hospital is finished, the clinical resources will be much greater no doubt. Lest it might be inferred that the delay in organizing the Medical Branch in Galveston has been the fault of the Regents, in light of their known opposition to a disintegration of the University, we will state, and the fact is recognized, and mentioned by the memorialists, that the Regents have clearly shown in their recent report, a copy of which is before us, that the present resources of the University are inadequate to erect the necessary buildings, equip and put in operation the school as it should be, (though the memorialists insist that the estimate on that head, $140,000 for buildings—is excessive; stating that those of the Medical Department of the University of Louisiana, with a capacity for 500 students, and including furniture, laboratory, museum and every appurtenance, cost just half that amount.) In order partly to meet the difficulty, and to hasten the consummation of the desired end, the city of Galveston offers to donate a block of ground, valued at $40,000 to $50,000 for the erection of the buildings; meantime, the late John Sealey left $50,000 for a hospital, and a condition to the offer is, that the hospital shall be located on the same block. The site offered is one of the most desirable in the city, and has never been overflowed. It commands a full view of the bay and the gulf, with the shipping, and every other point of interest within the harbor. It is accessible by street cars, yet sufficiently remote from the business center to be free from the noise and dust, &c. of the city.

The memorialists then treat the subject from the standpoint of economy, and show that every year, 200 Texas students carry $100,000 out of the State for medical instruction, which should be afforded them by the State. In this estimate our friends are very moderate, and fall far short of the actual figures; for the census of 1881 showed that with a population, then, of 1,500,000, Texas furnished 252 "regular" and 4 "irregular" medical students, and had 3003 physicians, (5th Illinois Report, S. B. H.) The population of the State is now certainly not short of 2,500,000; a corresponding ratio of increase in the number of students would give at present something over 400, with 5000 doctors; therefore the loss to the State, occasioned by the delay in putting the Medical Department into operation, may be put down at double the estimated sum, or, in round numbers, $200,000; or a sum sufficient to build and equip the college, and to "run it" for several years.

In light of these facts and figures, it is to be hoped that our legislators, who are such sticklers for economy, will not turn a deaf ear to the petition of the memorialists. In conclusion we will say that the subject is forcibly, elegantly and exhaustively presented, and the memorial does credit to the distinguished gentlemen comprising the commission. The legislators have already made one serious mistake in this connection—refused to give the University Regents control of the University lands. Had the lands been placed in the hands of the Regents, no doubt the consummation of a medical department would have been a matter of a few years. As it is, whether the college be located at Galveston, or incorporated into the University, we do not want to see any haste in the matter, nor action taken until the means at hand are adequate to put in operation the very highest order of a medical school.

